# Spectrofluorimetric Determination of Labetalol Hydrochloride in Pharmaceutical Preparations and Urine Samples

**Published:** 2008-06

**Authors:** Nafisur Rahman, SK Manirul Haque

**Affiliations:** *Department of Chemistry, Aligarh Muslim University, Aligarh-202002 (U.P.), India*

**Keywords:** labetalol, zinc (II) sulphate, eosin, spectrofluorimetry

## Abstract

Two simple and sensitive spectrofluorimetric methods have been developed for the determination of labetalol (LBT). In method A, the native fluorescence was measured at 432 nm after excitation at 312 nm. The second method (method B) is based on the formation of a ternary complex between zinc (II), eosin and LBT. The fluorescence intensity of the ternary complex was measured at 452 nm after excitation at 317 nm. Optimum conditions for the determination were also investigated. The linear range and detection limit for method A and B were found to be 1.25-30 µg/ml; 0.24 µg/ml and 0.5-4 µg/ml; 0.08 µg/ml, respectively. The proposed methods are simple, practical and relatively free of interference from coexisting substances. The methods have been applied to assess LBT in commercial tablets and human urine samples with good precision and accuracy.

## INTRODUCTION

Labetalol hydrochloride is chemically described as 5-[1-Hydroxy-2-(1-methyl-3-henyl propyl amino) ethyl] salicylamide hydrochloride. Labetalol (LBT) is a mixed antagonist i.e. α_1_ and non-selective β-receptor antagonist. It is used in the management of hypertension. After oral administration it is well absorbed and extensively metabolized in the liver. Metabolites are excreted in the urine together with small amounts of unaltered LBT. British Pharmacopoeia (1) has recommended non-aqueous titration for its determination whereas United States Pharmacopoeia (2) recommended HPLC method for LBT determination.

The therapeutic importance of the drug has engendered development of assays for its quantitation in commercial dosage forms and biological fluids. A literature survey revealed that analytical techniques including spectrophotometry (3-5), HPLC (6-9), HPLC-MS (10), micellar liquid chromatography (11), capillary liquid chromatography (12) capillary electrophoresis (13-14), capillary isotachophoresis (15) and NMR spectroscopy (16) have been employed for the determination of LBT. LBT was also analyzed in pharmaceutical preparations using an ion-selective electrode method (17). The determination in biological fluids normally requires the use of trace analysis techniques such as HPLC, LC, CE, cyclic voltametry, LC-MS, GC-MS, inductively coupled plasma-mass spectrometry. All of these methods are very expensive. In addition, these methods require long and tedious pretreatment of the samples and laborious clean up procedures prior to analysis. UV-visible spectrophotometry and fluorimetry are the techniques of choice in research laboratories, hospitals and pharmaceutical industries due to its low cost and inherent simplicity. Spectrofluorimetric methods have also been reported (18) for determination of LBT in commercial dosage forms and spiked human urine based on its interaction with ethylacetoacetate in presence of sulphuric acid and reaction between nitroso-derivative of LBT and 2-cyanoacetamide in the presence of ammonia (19).

This paper reports two simple, sensitive and accurate spectrofluorimetric methods for the determination of LBT. The first method is based on the direct measurement of the native fluorescence of the drug at 432 nm after excitation at 312 nm. In the second method, the ternary complex formed between zinc (II), eosin and labetalol was extracted into chloroform and its fluoresence intensity was measured at 452 nm after excitation at 317 nm. The proposed methods were extended to the determination of LBT in pharmaceutical preparations and human urine.

## EXPERIMENTAL

### Apparatus

All fluorescence measurements were performed using a ‘Hitachi F-2500 fluoresence’ spectrophotometer (Tokyo, Japan) equipped with a xenon lamp. Slit widths for both excitation and emission monochromators were set at 5 nm and all measurements were made in quartz cells with path length of 1.0 × 1.0 cm. Absorbance measurements were made on Elico UV-Visible Spectrophotometer, model No SL-164 (India).

**Materials and reagents**

- Reference standard sample of labetalol hydrochloride was obtained from Sigma Chemical Co, USA.
- Pharmaceutical preparations of labetalol such as lobet 100 (Samarth Pharma., India), gravidol 100 (Mercury Lab., India) were purchased from local market.
- Eosin (Fluka Chemie AG, Switzerland) solution was prepared as 2.0 ×10^-3^ M solution in distilled water.
- Zinc (II) sulphate (Sigma Aldrich Chemie, Germany) solution was prepared as 2.0 × 10^-3^ M solution in distilled water.
- Urine samples were obtained from healthy volunteers.
- Carbonate buffer of pH 9.4 was prepared by dissolving 26.5 g sodium carbonate and 21.0 g sodium bicarbonate in 500 ml distilled water.

**Standard LBT solution.** A stock solution of LBT (0.25 mg/ml) was prepared by dissolving 25 mg LBT in distilled water in 100 ml volumetric flask. The stock solution of LBT (0.25 mg/ml) was used to prepare working solutions by suitable dilutions with distilled water. The stock solution of LBT was stable at least 10 days at room temperature.

## METHODS

### Procedure for determination of LBT

**Method A.** Aliquots of stock solution (0.25 mg/ml) were transferred into a set of 10 ml volumetric flasks and volumes were completed to the mark with distilled water to produce solutions in the concentration range 1.0-30.0 µg/ml. Fluorescence intensities were measured at 432 nm after excitation at 312 nm. Calibration graphs were constructed by plotting fluorescence intensity against the final concentration of LBT.

**Method B.** Aliquots of stock solution (0.25 mg/ml) were pipetted into a series of 25 ml volumetric flasks to produce working solutions in the concentration range 0.5 to 4.0 µg/ml. To each flask, 0.8 ml of 2.0 × 10^-3^ M eosin and 0.7 ml of 2.0 × 10^-3^ M zinc (II) sulphate were added and diluted to volume with distilled water. The contents of the flask were transferred into a separating funnel and extracted with 25 ml chloroform by shaking for 1 min. The fluorescence intensity of dried organic layer was measured at 452 nm after excitation at 317 nm. Calibration graphs were obtained by plotting the fluorescence intensity versus the final concentration of LBT.

**Procedure for commercial tablets.** Five tablets (claming 100 mg of LBT per tablet) were accurately weighed and finely powdered. A quantity of the powder equivalent to 25 mg of LBT was extracted by shaking with 20 ml of distilled water, followed by another two extractions each with 10 mL of distilled water. It was filtered on Whatmann filter paper No. 42 (Whatmann International Limited, Kent, UK) to remove insoluble materials. The volume of filtrate was diluted to 100 ml with distilled water. It was further diluted according to the need and then analyzed following the proposed procedures. The nominal content of the tablets was calculated either from the previously plotted calibration graphs or using regression equations.

**Procedure for spiked human urine samples.** Aliquot volumes of spiked human urine samples were transferred into small separating funnel. 5 ml of carbonate buffer pH -9.4 was added and the solution was mixed well. The solution was then extracted with 3 × 5 ml of diethyl ether. The ether extract was collected and evaporated. The residue was dissolved in 5 ml of distilled water and the above general procedure was then followed. The nominal content of LBT was determined from the corresponding regression equation.

**Evaluation of bias.** The bias has been evaluated by means of point and interval hypothesis tests (20-21). In interval hypothesis the proposed method (method A & B) is accepted when the true mean is within ± 2% of that of the reference method, i.e.

$0.98<\mu_{2}/\mu_{1}<1.02$

which can be generalized to

$\theta_{L}<\mu_{2}/\mu_{1}<\theta_{u}$

where θ_L_ and θ_U_ are lower and upper acceptance limits, respectively. The limits of this confidence interval can be calculated using the following quadratic equation:

$\theta^{2}\left(\bar{x_{1}^{2}}-S_{p}^{2}t^{2}/n_{1}\right)-2\theta \bar{x_{1}}\bar{x_{2}}+\left(\bar{x_{2}^{2}}-S_{p}^{2}t^{2}/n_{2}\right)=0$

## RESULTS AND DISCUSSION

**Figure 1:**
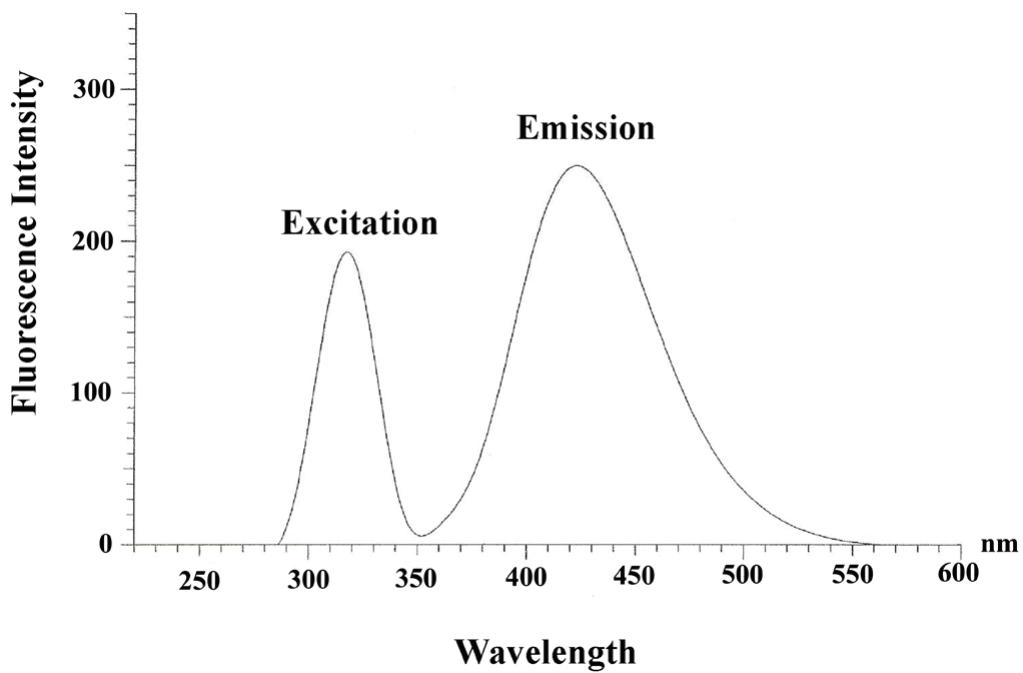
Excitation and emission spectra of 1.37 ×10^-5^ M aqueous solution of LBT.

Aqueous solution of LBT fluoresces at 432 nm with an excitation wavelength at 312 nm (Fig. 1). Based on this inherent fluorescence property, direct determination of LBT was achieved in pharmaceutical preparations and in urine samples.

**Figure 2:**
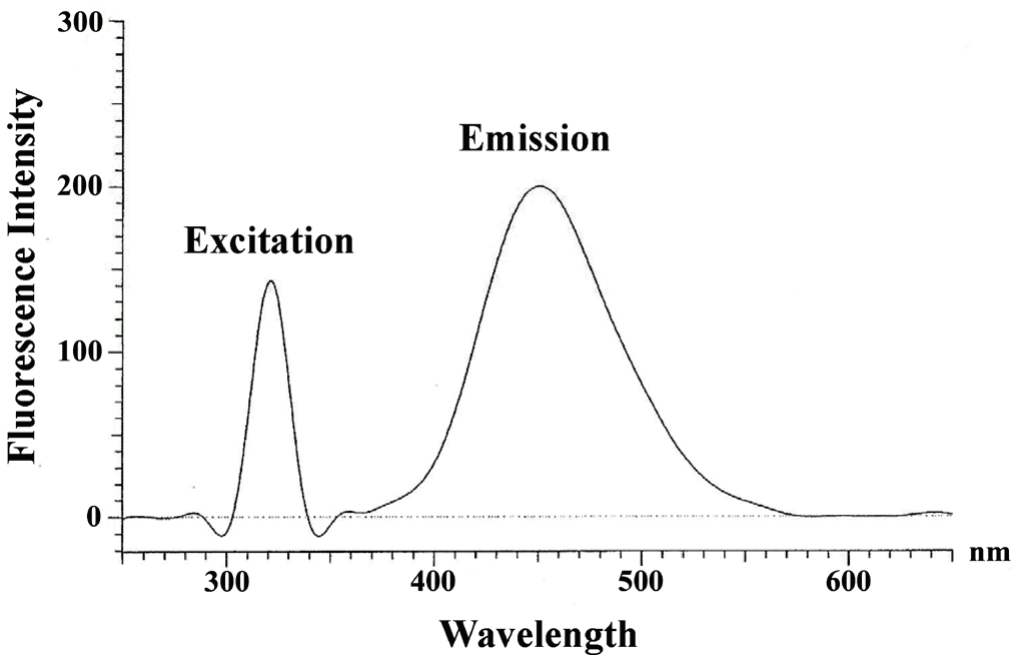
Excitation and emission spectra of ternary complex (0.4 ml of 6.85 × 10^-4^ M LBT + 0.8 ml 2.0 × 10^-3^ M Zn (II)) and 0.7 ml 2.0 × 10^-3^ M eosin extracted into 25 ml chloroform.

In the literature, spectrophotometric (22) and fluorimetric (23) methods have been reported based on the ternary complexes of general formula (L_n_M_x_S_y_). In the present study, a ternary complex involving LBT, Zn (II) and eosin was formed in which the main ligand L is LBT, the second ligand S is eosin and M is zinc (II). The ternary complex was extracted into chloroform which fluoresces at 452 nm after excitation at 317 nm (Fig. 2). The mixtures of LBT - eosin and LBT - Zn (II) were shaken, separately, with chloroform, and the organic layer, in both cases, did not exhibit fluorescence under the specified experimental conditions whereas the chloroformic extract of eosin - Zn (II) fluoresces at 655 nm after excitation at 327 nm. Therefore, it is concluded that interaction of Zn (II), eosin and LBT resulted in the formation of ternary complex.

### Optimization of reaction conditions

Investigations were carried out to establish the optimum reaction conditions with respect to reaction time, reagents concentration and shaking time for extraction.

**Effect of time.** The effect of time on the development of fluorophore and its stability was investigated. The reaction product got stabilized immediately after mixing LBT and the reagents and remained stable for at least 2 h.

**Effect of zinc (II) sulphate concentration.** The effect of volume of 2.0 × 10^-3^ M Zn (II) on the fluorescence intensity of the ternary complex was studied over the volume range 0.015-0.9 ml; keeping the concentrations of the drug (10 µg/ml) and eosin (0.8 ml of 2.0 × 10^-3^ M) constant. It was found that the maximum and constant fluorescence intensity was obtained over the range 0.5 - 0.9 ml of 2.0 × 10^-3^ M Zn (II). Thus, 0.7 ml of 2.0 × 10^-3^ M Zn (II) was used as an optimum volume for further measurement.

**Effect of eosin concentration.** In order to examine the effect of the volume of 2.0 × 10^-3^ M eosin on the ‘fluorescence intensity’, the reaction was carried out using different volumes (0.05-1.0 ml). The maximum and constant ‘fluoresence intensity’ was obtained at 0.8 ± 0.2 ml. Therefore, 0.8 ml of 2.0 × 10^-3^ M eosin was taken as the optimum volume for the assay procedure.

**Effect of shaking time for extraction.** In order to examine the effect of shaking time for the extraction of fluorophore into chloroform, experiments were performed for the periods ranging from 1-3 min. Maximum and constant fluorescence intensity was obtained by one minute of shaking and therefore, two minutes shaking time was recommended for the extraction of fluorophore.

### Validation of proposed methods

Under the optimum experimental conditions, the fluorescence intensity - concentration plots for methods A and B were found to be rectilinear over the range 1.25-30 µg/ml and 0.5-4 µg/ml, respectively. Linear regression analysis of calibration data gave the regression equations cited in Table 1 with correlation coefficients close to unity in both the cases. Statistical analysis of regression lines were made regarding the standard deviation of residuals (S_x/y_), standard deviation of slopes (S_b_) and standard deviation of intercepts (S_a_) and the values are summarized in Table 1. In both the spectroflourimetric methods, the values are small confirming that the proposed methods are precise (24). The LOD values for methods A and B were found to be 0.24 and 0.08 µg/ml, respectively.

**Table 1:** Analytical characteristic of the proposed methods

Parameters	Method A	Method B

Beer’s law limit (μg/ml)	1.25 - 30	0.5 - 4
Linear regression equation a	F =1.45 + 9.99 C	F = 10.95 ×10^-2^ + 25.49 C
S_a_	0.44	0.38
tS_a_^b^	9.74 ×10^-1^	8.51 ×10^-1^
S_b_	2.59 ×10^-2^	1.52 ×10^-1^
tS_b_^b^	5.77 ×10^-2^	3.39 ×10^-1^
Correlation coefficient (r)	0.9999	0.9998
Variance (S_x/y_^2^)	0.54	0.35
Detection limit (μg/ml)	0.24	0.08
Quantitation limit (μg/ml)	0.73	0.23
Recovery ± SD	100.25 ± 0.73	100.02 ± 0.59

a With respect to F = a + bC, where C is the concentration in μg/ml, F is fluorescence intensity;

b Confidence interval of the intercept and slope at 95% confidence level and ten degrees of freedom (t=2.228).

**Precision.** The within day precision assays were carried out through replicate analysis (n=5) of LBT corresponding to 5, 15 and 25 µg/ml for method A and 1.0, 2.6 and 4.0 µg/ml for method B. The interday precision was also evaluated through replicate analysis of the pure drug samples for five consecutive days at the same concentration levels as used in within day precision. The results of these assays are reported in Table 2. As can be seen from the Table 2 that RSD values for within day precision were always lower than 1.4% for method A and 2.8% for method B; RSD values for interday precision were lower than 1.1% for method A and 2.95% for method B. The precision results are satisfactory.

**Table 2:** Test of precision of the proposed methods

Proposed methods	Concentration (μg/ml)		RSD^a^ (%)	SAE^b^	C.L.^c^
	Taken	Found ± SD^a^			

Method A					
Intraday assay	5.00	5.062 ± 0.066	1.31	0.030	0.082
	15.00	15.053 ± 0.068	0.45	0.030	0.084
	25.00	25.042 ± 0.083	0.33	0.037	0.103
Interday assay	5.00	4.965 ± 0.050	1.01	0.022	0.062
	15.00	14.980 ± 0.083	0.56	0.037	0.104
	25.00	25.007 ± 0.182	0.73	0.082	0.226
Method B					
Intra day assay	1.00	0.998 ± 0.027	2.71	0.012	0.034
	2.60	2.602 ± 0.023	0.87	0.010	0.028
	4.00	4.007 ± 0.033	0.83	0.015	0.041
Inter day assay	1.00	1.003 ± 0.030	2.94	0.013	0.037
	2.60	2.604 ± 0.025	0.95	0.011	0.031
	4.00	4.006 ± 0.040	1.00	0.018	0.050

a Mean for five independent determinations;

b SAE, standard analytical error;

c C.L., confidence limit at 95% confidence level and four degrees of freedom (t = 2.776).

**Accuracy.** Method A was used for estimation of LBT from tablets after spiking with 100, 300 and 500% of additional pure drug. In case of method B, LBT was determined from tablets after spiking with 50, 150 and 250% of additional pure drug. The results are reported in Table 3. As can be seen from the Table 3 that recoveries ranged from 99.99-100.13% for method A and 99.87-100.16% for method B. The selectivity of the proposed methods was ascertained by analyzing standard LBT in the presence of tablet excipients such as lactose, starch, glucose, cellulose, talc and magnesium stearate. It was observed that these excipients did not interfere with the proposed methods.

**Table 3:** Accuracy and recovery

Formulation	Excess of the drug added (%)	Recovery ± RSD (%)	SAE	CL

Method A^a^				
Lobet 100	100	100.129 ± 1.16	0.052	0.144
	300	100.008 ± 0.45	0.041	0.112
	500	99.992 ± 0.26	0.035	0.097
Gravidol 100	100	100.033 ± 1.53	0.068	0.190
	300	99.993 ± 0.70	0.063	0.175
	500	100.027 ± 0.68	0.091	0.253
Method B^b^				
Lobet 100	50	100.162 ± 1.60	0.011	0.030
	150	100.061 ± 1.21	0.014	0.037
	250	100.052 ± 1.18	0.019	0.051
Gravidol 100	50	100.084 ± 1.65	0.011	0.031
	150	100.096 ± 1.14	0.013	0.035
	250	99.874 ± 0.99	0.016	0.043

a Initial amount taken=5.0 μg/ml;

b Initial amount taken=1.0 μg/ml.

The proposed methods were applied to the determination of LBT in its commercial tablets. The results of the proposed methods were compared with those obtained by the reference method (25) (Table 4). Statistical analysis of the results using Student’s t-test and variance ratio F-test revealed no significant difference between the proposed methods (Method A and B) and the reference method at 95% confidence level regarding accuracy and precision.

**Table 4:** Assay results of LBT in commercial tablets using the proposed methods and reference method

Formulations	Method A	Method B	Reference Method (25)

Lobet 100 tablets			
Recovery (%)	100.12	100.110	100.23 99.62
RSD	1.470	0.843	1.19 1.74
t	1.205	1.418	
F	1.519	4.191	
θ_L_	0.998	0.990	
θ_U_	1.004	1.101	
Gravidol 100 tablets				
Recovery (%)	100.010	100.13	100.18 99.91
RSD	1.300	1.140	1.38 2.55
t	2.201	0.437	
F	1.043	3.717	
θ_L_	0.999	0.996	
θ_U_	1.004	1.000	

The bias of each drug sample was also checked based on recovery experiments using interval hypothesis test. As can be seen from Table 4 that the true bias of all samples is lower than ± 2% confirming that there is no significant differences between the proposed method and reference method with regard to accuracy and precision at 95% confidence level.

The performance of the proposed methods was compared with other existing spectrofluorimetric methods (18, 19). In case of the proposed method B and reported method (18), the products are formed immediately after mixing the reagents but the precision of the reported method is somewhat poor with relatively higher LOD value. However, the LOD value of the other method (19) is very small but the drawback is that it requires 25 min of heating for reaction to complete. Therefore, the proposed methods were found to be simple and can compete with existing spectrofluorimetric methods in determining the drug in pharmaceutical preparations.

The proposed methods were further extended to the *in vitro* determination of LBT in spiked human urine samples. In hypertensive patients, LBT is orally given in doses of 100 mg three times daily and consequently, it results in urine level at concentration level of about 2-4 µg/ml. This concentration fell well within working range of proposed methods. The calibration graphs were first constructed by plotting fluorescence intensity vs. increasing concentration of LBT in spiked human urine samples over the concentration ranges 1.25-30 µg/ml for method A and 0.5-4.0 µg/ml for method B. These results (Table 5) are satisfactorily accurate and precise.

**Table 5:** Application of the proposed spectrofluorimetric methods to the determination of LBT in spiked human urine

	Amount added (μg/ml)	Amount found (μg/ml)	Recovery (%)

Method A	1.0	0.974	97.41
	2.0	2.081	100.05
	3.0	2.993	99.78
	4.0	3.995	99.88
	5.0	5.025	100.51
	x		100.33
	RSD		2.39
Method B	1.6	1.625	101.56
	2.0	1.990	99.50
	2.4	2.435	101.46
	2.8	2.755	98.39
	x		100.84
	RSD		1.15
